# Comparative genomics across *Linum* illuminates the evolutionary basis of flax domestication, genome diversification, and seed oil biosynthesis

**DOI:** 10.3389/fgene.2026.1835945

**Published:** 2026-06-16

**Authors:** Liuxi Yi, Rula Sa, Yingnan Mu, Jinhao Zhang, Ruichao He, Yadi Song, Yu Zhou, Yongsheng Chen

**Affiliations:** 1 College of Agriculture, Inner Mongolia Agricultural University, Hohhot, China; 2 College of Grassland Science, Inner Mongolia Agricultural University, Hohhot, China; 3 Inner Mongolia Academy of Agricultural and Animal Husbandry Sciences, Hohhot, China

**Keywords:** comparative genomics, crop domestication, fatty acid desaturase, gene family, *Linum*, LTR retrotransposon, NLR

## Abstract

Flax (*Linum usitatissimum* L.) is a globally significant dual-purpose crop cultivated for both bast fiber and seed oil, yet the genomic basis of its diversification and domestication from wild relatives remains incompletely understood. In this study, we performed a comprehensive comparative genomic analysis of 11 *Linum* accessions-including five cultivated flax accessions and their wild progenitor *Linum bienne*-alongside two outgroups (*Populus trichocarpa* and *Erythroxylum novogranatense*). To reduce annotation heterogeneity, we uniformly re-annotated all genomes using the deep-learning-based ANNEVO pipeline, which reduced apparent gene fragmentation and maintained broadly comparable annotation completeness under a unified framework. Phylogenomic analysis resolved a pre-domestication macro-evolutionary gene family expansion in the *L. bienne*-*L. usitatissimum* lineage, providing standing genetic variation that subsequently diverged into pathways enriched for cell wall biogenesis in fiber flax and lipid/photosynthetic metabolism in oil flax. Genome-wide NLR analysis revealed a globally contracted immune receptor repertoire in *Linum* and quantitative stability between cultivated flax and *L. bienne*, while chromosomal distribution and synteny analyses indicated that a major NLR-rich region differs in chromosome assignment between *L. bienne* and cultivated flax rather than providing definitive evidence for *de novo* cluster gain or complete cluster loss. Furthermore, genome size variation across *Linum* was predominantly driven by transposable elements, with remarkably recent bursts of long terminal repeat (LTR) retrotransposons (<0.5 Mya) shared by cultivated flax and L. bienne but clearly predating human domestication, indicating an inherited repeat landscape rather than a domestication-associated event. Finally, analysis of fatty acid desaturases revealed striking evolutionary asymmetry: the FAD3 family remained highly conserved, whereas FAD2 exhibited exceptional plasticity, characterized by lineage-specific tandem expansions and a putative large structural absence associated with the contracted FAD2 repertoire in the oil flax accession T397. Together, these findings provide a standardized genomic framework for *Linum* and illuminate the structural variations, repeat dynamics, and pre-adaptive gene duplications that may have contributed to flax domestication and agronomic specialization.

## Introduction

Flax (*Linum usitatissimum* L.) is one of the foundational founder crops of early agriculture, possessing a dual agricultural identity as both a premier source of bast fibers for textiles and a vital oilseed crop (Allaby et al., 2005; Zohary et al., 2012). Cultivated flax was domesticated from its wild progenitor, *Linum bienne*, approximately 8,000 to 10,000 years ago (Fu, 2011; Soto-Cerda et al., 2013). Over millennia of human selection, flax diverged into distinct morphotypes: fiber flax, characterized by tall, unbranched stems and robust secondary cell wall deposition, and oil (linseed) flax, optimized for profuse branching, high seed yield, and the accumulation of polyunsaturated fatty acids, notably α-linolenic acid (Green, 1986; Zhang et al., 2020; Lu et al., 2025). Despite the economic and historical significance of flax, the comprehensive genus-wide genomic events that facilitated this phenotypic radiation and the broader evolutionary history of the *Linum* genus (McDill et al., 2009; Ruiz-Martín et al., 2018) remain incompletely resolved.

The recent proliferation of high-quality, chromosome-scale genome assemblies for cultivated flax accessions (You et al., 2018; Sa et al., 2021; Lu et al., 2025; Yadav et al., 2025) and diverse wild *Linum* species (Gutiérrez-Valencia et al., 2022; 2024; Innes et al., 2023; Zervakis et al., 2025) provides an unprecedented opportunity to investigate domestication at the genus level. However, cross-species comparative genomics is frequently confounded by severe inconsistencies in genome annotation (Salzberg, 2019). Publicly available genomes are often annotated utilizing disparate pipelines, varying standards of RNA-seq evidence, and divergent parameters, which systematically introduce artifacts such as artificial gene fragmentation or over-prediction (Stanke et al., 2006; Campbell et al., 2014). Such biases critically distort downstream analyses of orthology, gene family expansion, and evolutionary dynamics, necessitating the implementation of unified, state-of-the-art re-annotation frameworks before robust biological inferences can be drawn (Manni et al., 2021).

Gene duplication and subsequent functional divergence are primary drivers of plant morphological and metabolic innovation (Lynch and Conery, 2000; Flagel and Wendel, 2009). During crop domestication, selection acts upon these genomic redundancies to shape agronomic traits (Doebley et al., 2006; Meyer and Purugganan, 2013). In flax, the disparate breeding objectives for fiber and oil morphotypes likely imposed divergent selection pressures on specific biological pathways, such as endomembrane trafficking for cell wall biosynthesis (Cosgrove, 2016) and photosynthetic carbon allocation for lipid accumulation (Baud and Lepiniec, 2009). Concurrently, transposable elements (TEs), particularly long terminal repeat (LTR) retrotransposons, exert profound influences on genome architecture, often dictating genome size and generating structural variations that facilitate crop adaptation (Feschotte et al., 2002; Lisch, 2013; Bennetzen and Wang, 2014).

Beyond developmental and metabolic traits, the evolutionary arms race with pathogens profoundly shapes plant genomes, primarily through the dynamic birth-and-death evolution of nucleotide-binding leucine-rich repeat (NLR) immune receptors (Michelmore and Meyers, 1998; Meyers et al., 2003), which often evolve through birth-and-death dynamics (Jones et al., 2016). The flax–flax rust (*Melampsora lini*) pathosystem is a historical paradigm for understanding gene-for-gene resistance (Flor, 1956; Dodds et al., 2006). However, how the spatial and quantitative architecture of the NLR repertoire evolved across the *Linum* genus and responded to the specific epidemiological pressures of agricultural domestication requires whole-genome spatial analysis (Van De Weyer et al., 2019).

Furthermore, the defining biochemical feature of flaxseed is its exceptional enrichment in polyunsaturated fatty acids, an attribute strictly governed by membrane-bound fatty acid desaturases (FADs). Specifically, FAD2 catalyzes the conversion of oleic acid to linoleic acid, while FAD3 orchestrates the subsequent conversion to α-linolenic acid (Arondel et al., 1992; Okuley et al., 1994). In flax, FAD3 genes have been directly linked to seed linolenic acid content (Vrinten et al., 2005), and recent genomic/transcriptomic analyses have further identified key FAD2, FAD3, and SAD genes involved in fatty acid biosynthesis (Dvorianinova et al., 2023). In various oilseed crops, FAD2 paralogues frequently exhibit dynamic copy number variations associated with environmental adaptation and artificial selection (Dar et al., 2017). Yet, the precise genomic mechanisms underlying the remodeling of the desaturase machinery across *Linum* species and within domesticated flax accessions remain elusive. To address these gaps, we conducted a systematic comparative genomic study utilizing 11 *Linum* genomes and two outgroups. By employing a unified re-annotation strategy, we investigated phylogenomic history, trait-specific gene family evolution, chromosome-level distribution and syntenic context of NLR clusters, LTR retrotransposon dynamics, and the structural evolution of FAD2 and FAD3.

## Methods

### Genomic data acquisition, assembly quality summary, and standardized re-annotation

Genome assemblies for 11 *Linum* accessions and two outgroup species were retrieved from public databases (Supplementary Table S1). The dataset included five *L. usitatissimum* accessions (three oil types: T397 [GCA_051167515.1] (Yadav et al., 2025), Marquise [GCA_052935155.1] (Demenou et al., 2025), Attila [GCA_052935085.1] (Demenou et al., 2025); two fiber types: Bolchoi [GCA_052935075.1] (Demenou et al., 2025), YY5 [Zenodo.4872894] (Sa et al., 2021)), and six wild *Linum* species: *Linum lewisii* (GCA_034768395.1, Innes et al., 2023), *Linum grandiflorum* (GCA_965225055.1, Zervakis et al., 2025), *L. bienne* (GCA_965366145.1), *Linum perenne* (GCA_965231395.1, Zervakis et al., 2025), *Linum tenue* (GCA_946122785.1, Gutiérrez-Valencia et al., 2022), and *Linum trigynum* (GCA_964030455.1, Gutiérrez-Valencia et al., 2024). *Populus trichocarpa* (GCA_000002775.4, Tuskan et al., 2006) and *Erythroxylum novogranatense* (GCA_029891385.1, Wang et al., 2023) were utilized as outgroups.

To document differences in genome assembly quality among species, we compiled assembly-level statistics for all genomes, including assembly level, BUSCO completeness, contig N50 and L50, scaffold N50 and L50, number of scaffolds, GC content, and assembled genome size (Supplementary Table S1). Contig/scaffold continuity statistics, scaffold number, GC content, and assembled genome size were calculated using QUAST v5.3.0 (Gurevich et al., 2013). Assembly completeness was evaluated using BUSCO v6 (Manni et al., 2021) with the eudicots_odb10 dataset. These metrics were used to evaluate potential assembly-related limitations in downstream chromosome-level and gene-family analyses.

To mitigate bias from heterogeneous original annotations, all genomes were uniformly re-annotated using the deep-learning-based *ab initio* annotation software ANNEVO v2.2 (Zhang et al., 2026). ANNEVO was run using default parameters with the ANNEVO_Embryophyta.pt model for all assemblies to ensure that gene prediction was performed under a consistent framework across species and accessions. No species-specific RNA-seq or homology evidence was incorporated during ANNEVO prediction, because comparable multi-tissue transcriptomic resources are not available for all genomes analyzed here. This design was intended to reduce evidence-availability bias among species, although it does not replace future fully evidence-guided annotation efforts. Annotation completeness was evaluated using BUSCO v6 with the eudicots_odb10 dataset. Only protein-coding gene models predicted by ANNEVO were used for downstream orthogroup clustering and gene family analyses.

### RNA-seq-based validation of YY5 gene annotations

To independently evaluate the quality of YY5 gene annotations, publicly available RNA-seq data from five tissues, including leaf, stem, seed, flower, and root, were used. RNA-seq reads from each tissue were first aligned to the YY5 reference genome using HISAT2 v2.2.2 (Kim et al., 2015). The resulting alignments were then assembled into transcripts using StringTie v3.0.3 (Pertea et al., 2015) for each tissue separately, and a merged transcript set was generated from the five tissue-specific assemblies. The original YY5 annotation and the ANNEVO annotation were compared with the RNA-seq-derived transcript evidence based on the longest transcript of each gene. Annotation quality was assessed using exon-base support ratio, RNA-seq exon-base capture ratio, intron support ratio, RNA-seq intron capture ratio, exact exon support ratio, intron-chain support ratio, and the proportion of annotated transcripts with ≥80% exon-overlap support. All RNA-seq libraries were treated as unstranded during the analysis.

### Gene family clustering, phylogenomics, and expansion/contraction analysis

The re-annotated protein sequences were subjected to orthologous group clustering and species tree construction using OrthoFinder v3.1.0 (Emms and Kelly, 2015). Because ANNEVO generated one representative protein-coding model per locus, all predicted proteins were used directly for orthogroup clustering. Single-copy orthologous genes identified by OrthoFinder were used for phylogenomic reconstruction. A total of 239 single-copy orthologous genes were retained for species-tree construction. The resulting species tree was converted into an ultrametric tree using r8s v1.81 (Sanderson, 2003). Divergence-time calibration was performed using established speciation nodes from the TimeTree database (Kumar et al., 2022). Gene family expansion and contraction across the evolutionary nodes were inferred using CAFE v4.2.1 (De Bie et al., 2006) based on the ultrametric tree and orthogroup counts. Functional annotations were generated using eggNOG-mapper v2 (Cantalapiedra et al., 2021). Gene Ontology (GO) enrichment analysis for specific gene families was executed using the clusterProfiler v3.10.1 (Wu et al., 2021) package in R.

### Annotation of NLR disease resistance genes

Genome-wide identification of nucleotide-binding leucine-rich repeat (NLR) genes was performed using NLR-Annotator v2.1b (Steuernagel et al., 2020) with default parameters. The identified NLRs were classified into structural subclasses (e.g., CNL, TNL, NL) based on their motif architectures. The chromosomal coordinates of the annotated NLRs were extracted, and spatial density distributions across pseudochromosomes were calculated and visualized to identify apparent presence-absence patterns and clustering events. To further evaluate whether the major reciprocal NLR-cluster pattern between *L. bienne* and cultivated flax reflected independent cluster gain/loss or larger-scale chromosomal correspondence, we performed pairwise synteny analysis between L. bienne and T397 using JCVI v1.6.4 (Tang et al., 2024) based on the standardized ANNEVO gene models.

### Transposable element annotation and LTR insertion dynamics

Whole-genome transposable elements (TEs) were identified and classified using the EDTA v2.2.2 (Ou et al., 2019; Su et al., 2021) pipeline. TE annotations were summarized by major repeat classes, including LTR retrotransposons, DNA transposons, and other repeat categories. The total TE span was calculated for each genome and compared with assembled genome size. The relationship between total TE content and genome size was evaluated using linear regression, and the coefficient of determination (*R* ^2^) and *P*-value were reported. For evolutionary timing, intact long terminal repeat (LTR) retrotransposons were specifically extracted, and their insertion ages were estimated using LTR_retriever v3.0.4 (Ou and Jiang, 2018), relying on sequence divergence between paired LTR sequences. LTR insertion-time distributions were used to compare relative timing of repeat activity among species. Because estimated insertion times depend on mutation-rate assumptions and intact LTR recovery, these values were interpreted as approximate temporal signals rather than exact dates.

### Identification and evolutionary analysis of FAD2 and FAD3 families

To annotate the fatty acid desaturase families, protein sequences were initially scanned using HMMER v3.3.2 (Eddy, 2011) against the PF00487 hidden Markov model (Mistry et al., 2021). Candidate membrane-bound desaturases were subsequently clustered with reference *Arabidopsis thaliana* desaturases (FAD2, FAD3, FAD6, FAD7, FAD8, FAH12, SLD1, SLD2, and ADS1) using FastTree v2.2.0 (Price et al., 2010) to separate major clades. The extracted FAD2 and FAD3 family members were then aligned, and Maximum-Likelihood (ML) phylogenetic trees were reconstructed using IQ-TREE v2.0.3 (Minh et al., 2020) with 1,000 bootstrap replicates. The resulting phylogenies were visualized utilizing the ggtree v3.6.0 (Xu et al., 2022) package in R. Intraspecific genomic collinearity and structural variations surrounding desaturase loci were investigated using JCVI v1.6.4 (Tang et al., 2024). To test whether the apparent absence of canonical FAD2 genes in *L. tenue* and *L. trigynum* could be caused by missed gene prediction, we additionally performed genome-level TBLASTN in NCBI BLAST + v2.14.1+ (Camacho et al., 2009) searches using the *Arabidopsis* FAD2 protein as query against the *L. tenue*, *L. trigynum*, and YY5 genome assemblies. YY5 was used as a positive control. TBLASTN hits were overlapped with GFF gene annotations, and the corresponding genes were compared with the PF00487-containing desaturase candidate lists. This analysis was used only as an annotation-independent check for missed FAD2-like genomic regions, whereas final FAD2/FAD3 classification remained based on reference-guided phylogenetic analysis.

### HiFi read-alignment validation of the T397 FAD2-containing structural absence

To evaluate whether the apparent absence of the FAD2-containing syntenic block in T397 could reflect an assembly gap, publicly available HiFi reads from YY5 (SRR14339745) and T397 (SRR29936750) were aligned to the YY5 reference genome using minimap2 v2.30-r1287 (Li, 2018) with the HiFi preset parameter -ax map-hifi. The resulting alignments were sorted using samtools sort v1.22.1 (Li et al., 2009). Read-depth patterns across the YY5 chromosome 14 FAD2-rich interval were then calculated using mosdepth v0.3.1 (Pedersen and Quinlan, 2018) with a minimum mapping quality threshold of 20 and filtering of secondary, supplementary, duplicate and QC-failed alignments (mosdepth -Q 20 -F 3844). T397 HiFi reads mapping to this YY5 interval were extracted from the alignment files and realigned to the T397 assembly using minimap2 with the same HiFi alignment setting (-ax map-hifi). The realigned BAM files were sorted with samtools sort and inspected to determine whether these reads originated from an unassembled T397 chromosome 14 segment or from homologous retained regions elsewhere in the T397 genome. This analysis provided read-level support for interpreting the absence of the FAD2-containing block in T397, while recognizing that additional targeted experimental validation would be required to confirm the precise structural breakpoint.

## Results

### Standardized genome re-annotation and quality assessment of the *Linum* genus

Accurate and consistent gene annotation is a fundamental prerequisite for comparative genomics. To comprehensively investigate the evolutionary dynamics of the flax genus (*Linum*), we retrieved genome assemblies for 11 *Linum* accessions and two outgroups (*Populus trichocarpa* and *Erythroxylum novogranatense*) from public databases (Supplementary Table S1). However, original gene counts varied substantially among available annotations, ranging from 34,572 to 49,616 among cultivated *L. usitatissimum* accessions, and original annotation files were unavailable for T397 and *L. bienne*. To reduce methodological heterogeneity and construct a robust comparative framework, we performed a unified, *ab initio* genome re-annotation for all 13 species using ANNEVO (Zhang et al., 2026), a deep-learning-based annotation tool capable of high-accuracy gene prediction without relying on species-specific RNA-seq evidence.

**FIGURE 1:**
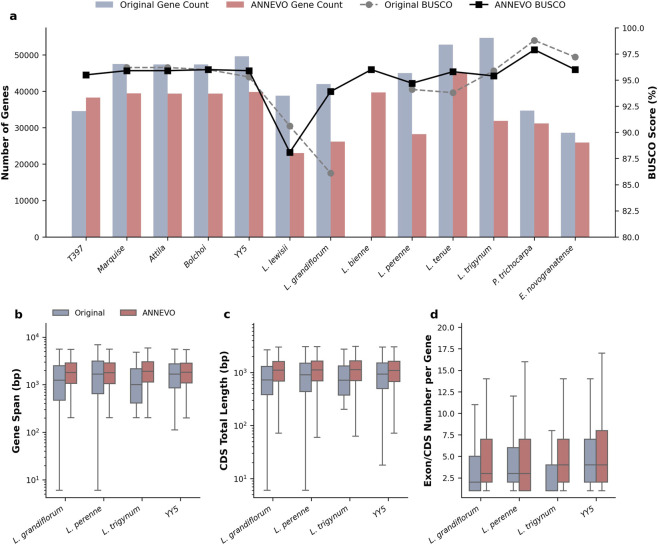
Standardized re-annotation of the *Linum* genus reduces apparent gene fragmentation while maintaining broadly comparable annotation completeness. **(a)** Comparison of total predicted gene counts and genome annotation completeness across 13 assemblies. Bar plots (left y-axis) show the number of predicted genes in the original annotations (grey) versus the ANNEVO re-annotations (red). Line plots (right y-axis) indicate the corresponding benchmarking universal single-copy orthologs (BUSCO) scores. Original BUSCO values for T397 and *L. bienne* are not shown because the corresponding original annotation files are not publicly available. **(b–d)** Structural comparisons of predicted gene models between the original and ANNEVO annotations for four representative genomes (*L. grandiflorum*, *L. perenne*, *L. trigynum*, and YY5). Box plots display the distributions of total gene span **(b)**, total CDS length **(c)**, and the number of exons/CDS per gene **(d)**. Data in **(b,c)** are plotted on a logarithmic scale (*log*_10_). In all box plots, the central horizontal line indicates the median, the box limits represent the first and third quartiles, and the whiskers extend to 1.5× the interquartile range. All structural metrics (gene span, CDS length, and exon count) in the ANNEVO annotations are significantly greater than those in the original annotations (*P* < 0.001, two-sided Mann-Whitney U test).

The standardized ANNEVO annotations yielded more compact gene sets than most original annotations while maintaining broadly comparable BUSCO completeness (Figure 1a). Across *Linum* genomes with available original BUSCO values, the average BUSCO score increased from 93.80% in the original annotations to 94.62% after ANNEVO re-annotation. In cultivated *L. usitatissimum* accessions with available original BUSCO values, ANNEVO retained the same average BUSCO completeness as the original annotations, despite a substantial reduction in gene number. Specifically, Marquise, Attila, Bolchoi, and YY5 had an average original BUSCO score of 95.93%, and the corresponding ANNEVO annotations also averaged 95.93%, while the average gene number decreased from approximately 47,973 to 39,481. Several genomes showed slightly lower BUSCO scores after re-annotation, such as Marquise, Attila, *L. lewisii*, *L. trigynum*, *P. trichocarpa*, and *E. novogranatense*. However, most of these decreases were small and should be interpreted together with the reduced gene number and structural support analyses, because BUSCO evaluates a conserved single-copy subset and does not fully capture whole-genome annotation redundancy, fragmentation, or exon-intron structure accuracy.

To further assess gene model structure, we compared original and ANNEVO annotations in representative genomes with available annotation files, including *L. grandiflorum*, *L. perenne*, *L. trigynum*, and YY5. ANNEVO-annotated genes generally showed longer gene spans, longer total CDS lengths, and higher exon/CDS counts per gene than the original annotations (Figures 1b–d; all *P* < 0.001, two-sided Mann-Whitney U test). For example, in YY5, the average CDS length increased from 1,150.1 bp in the original annotation to 1,296.5 bp in the ANNEVO annotation, while the average number of CDS per gene increased from 5.3 to 5.6. These patterns suggest that the reduced gene number in the ANNEVO annotations is associated with more compact and structurally complete gene models rather than a simple loss of coding sequences.

We further performed an independent RNA-seq-based validation using YY5 transcriptome data from five tissues: leaf, stem, seed, flower, and root (Supplementary Table S2). The original YY5 annotation and the ANNEVO annotation were evaluated against RNA-seq-derived transcript structures. Although ANNEVO predicted fewer genes than the original YY5 annotation, its gene models showed stronger RNA-seq support across multiple structural metrics. Using the merged five-tissue RNA-seq evidence, ANNEVO increased the exon-base support ratio from 84.61% to 90.39%, the intron support ratio from 83.48% to 84.57%, the exact exon support ratio from 53.22% to 58.25%, and the intron-chain support ratio from 49.17% to 51.95%. Moreover, the proportion of annotated transcripts with ≥80% exon-overlap support increased from 75.09% in the original annotation to 86.45% in the ANNEVO annotation. Across individual tissues, ANNEVO also showed consistently higher average support ratios for exon bases, introns, exact exons, intron chains, and transcript-level exon overlap. Together, these results indicate that ANNEVO generated more compact gene models with broadly comparable BUSCO completeness and improved RNA-seq structural support in YY5.

### Phylogenomic relationships and lineage-associated gene family dynamics in *Linum*

**FIGURE 2:**
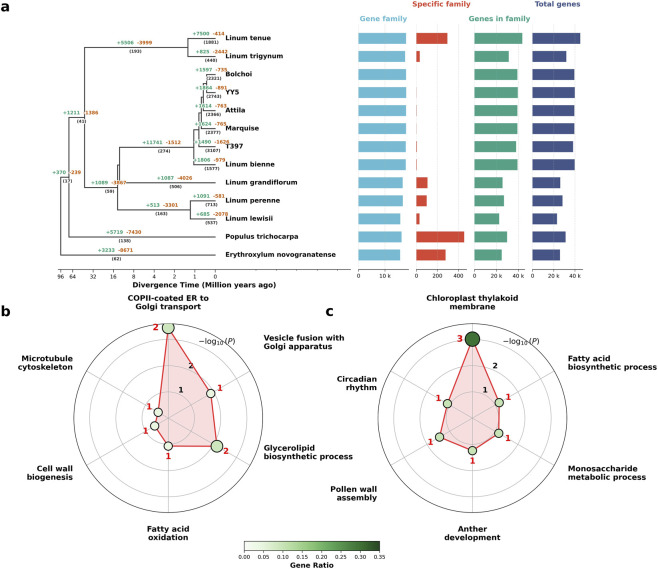
Phylogenomic evolution and functional divergence of lineage-specific gene families in *Linum*. **(a)** Maximum-likelihood phylogenetic tree, divergence times, and gene family dynamics of 11 *Linum* accessions and two outgroups (*Populus trichocarpa* and *Erythroxylum novogranatense*). The phylogenetic tree was reconstructed using 239 single-copy orthologous genes identified by OrthoFinder. Branch lengths indicate divergence time (Million years ago, Mya) estimated via molecular clock analysis. The green numbers with a plus sign (+) and orange numbers with a minus sign (−) on the branches represent the number of expanded and contracted gene families, respectively, inferred by CAFE analysis. Numbers in parentheses indicate rapidly evolving gene families. The right panel displays bar charts detailing the total number of gene families (light blue), species/lineage-specific gene families (orange), total genes clustered in families (green), and the total number of predicted genes (dark blue) for each genome. **(b,c)** Comparative Gene Ontology (GO) enrichment analysis of candidate lineage-associated orthogroups identified in fiber flax **(b)** and oil flax **(c)**. The radar charts display representative biological processes shaping the distinct agronomic traits of the two morphotypes. In both panels, the radial axis (distance from the center) represents the statistical significance (−*log*_10_ *P*-value) of the enrichment. The size of the circular nodes is proportional to the number of specific genes enriched in that category (also denoted by the red numbers adjacent to each node). The color gradient of the nodes reflects the Gene Ratio (the proportion of genes of interest relative to the background in a given GO term), with darker green indicating a higher ratio. Notably, the candidate fiber-flax-associated gene set **(b)** exhibits profound enrichment in endomembrane trafficking and cell wall biogenesis, consistent with processes involved in secondary cell wall deposition. In contrast, oil flax **(c)** is preferentially enriched in photosynthetic light harvesting, fatty acid biosynthesis, and reproductive organ development, consistent with possible breeding-associated emphasis on carbon assimilation, reproductive development, and seed oil yield.

To elucidate the evolutionary history and genomic diversification of the genus *Linum*, we reconstructed a whole-genome phylogenetic tree using 239 single-copy orthologous genes from the 11 *Linum* genomes and two outgroups. Phylogenomic analysis resolved the evolutionary relationships with high confidence, revealing that the *Linum* common ancestor diverged approximately 41.26 million years ago (Mya) (Figure 2a). The genus subsequently split into two major clades: one comprising *L. tenue* and *L. trigynum*, and a larger clade encompassing the remaining wild species alongside the cultivated morphotypes. Notably, the cultivated flax varieties (*L. usitatissimum*) exhibit an extremely close phylogenetic affinity to their wild progenitor, *L. bienne*, with their divergence occurring recently (∼1.05 Mya). Within the *L. usitatissimum* clade, the fiber-type and oil-type cultivars formed distinct monophyletic subclades.

Genome size and gene repertoire exhibit substantial variation across the genus, reflecting dynamic evolutionary trajectories. The total number of predicted protein-coding genes ranges from 23,034 in *L. lewisii* to 44,993 in *L. tenue*, with the latter also harboring the highest number of species-specific gene families (295) (Figure 2a). Crucially, analysis of Computational Analysis of gene Family Evolution (CAFE) inferred a striking macro-evolutionary genomic event: a large-scale gene family expansion (11,741 expanded vs. 1,512 contracted) occurred at the ancestral node shared by *L. usitatissimum* and *L. bienne* (Figure 2a). This expansion was inferred at a node predating the divergence between cultivated flax and *L. bienne*.

To explore candidate genomic signals associated with the divergent domestication trajectories of fiber and oil flax, we interrogated their candidate lineage-associated gene families. We identified 40 and 21 gene families putatively retained or expanded in fiber and oil flax subclades, respectively (the majority being predominantly single-copy). Functional enrichment analysis of these candidate lineage-associated gene sets revealed highly distinct biological themes that are broadly consistent with their respective agronomic traits (Figures 2b,c). In fiber flax, candidate lineage-associated genes are significantly enriched in vesicle-mediated transport pathways (e.g., COPII-coated ER to Golgi transport vesicles) and cell wall biogenesis (Figure 2b; Supplementary Table S3). Conversely, candidate oil flax-associated genes exhibit significant enrichment in photosynthesis-related components (e.g., chloroplast thylakoid membrane), light-harvesting, and fatty acid biosynthetic processes (Figure 2c; Supplementary Table S4). Oil flax-associated genes were also enriched in terms related to floral organ and pollen wall development.

### Evolutionary contraction and chromosome-level redistribution of the flax NLR repertoire

**FIGURE 3:**
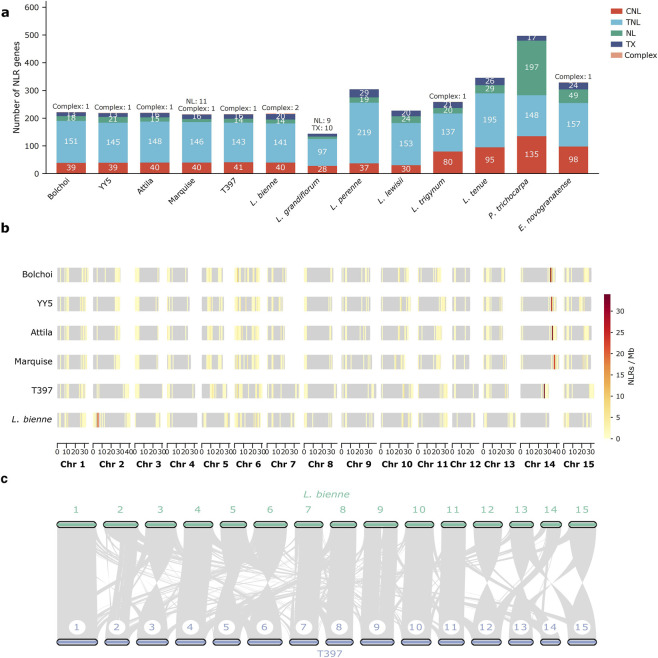
Evolutionary dynamics and chromosomal redistribution of NLR-rich regions in *Linum*. **(a)** Classification and quantitative distribution of NLR genes across 13 genomes, including cultivated flax, wild *Linum* accessions, and outgroups (*P. trichocarpa* and *Erythroxylum novogranatense*). Colors indicate distinct NLR subclasses (CNL, TNL, NL, TX, and Complex). Total gene counts and subclass compositions are detailed within and above the bars, highlighting the genus-level contraction in *Linum* and the quantitative conservation during flax domestication (from *L. bienne* to cultivated accessions). **(b)** Chromosomal distribution and density of NLR genes across the 15 chromosomes of five cultivated flax accessions and their wild progenitor, *L. bienne*. Heatmap colors represent the density of NLRs per megabase (Mb). The major NLR cluster is located on chromosome 2 in *L. bienne* but on chromosome 14 in cultivated flax accessions, producing an apparent reciprocal cluster-level difference. **(c)** Whole-genome synteny between *L. bienne* and T397 generated using JCVI. Grey ribbons connect collinear genomic blocks; *L. bienne* chromosomes are shown at the top and T397 chromosomes at the bottom. The syntenic correspondence between *L. bienne* chromosome 2 and T397 chromosome 14 indicates that the major NLR clusters in these chromosomes occur within collinear genomic contexts.

Genome-wide annotation of nucleotide-binding leucine-rich repeat (NLR) receptors across 13 genomes revealed a distinct evolutionary trajectory for the *Linum* (flax) genus. Compared to outgroup species, most *Linum* genomes contained fewer NLR genes and showed a TNL-biased subclass composition. (Figure 3a; Supplementary Table S5). For instance, the outgroup *Populus trichocarpa* (*Ptri*) maintains an expansive NLR array of 497 genes—characterized by a massive expansion of truncated NLs (197) and a balanced CNL-to-TNL ratio. In contrast, all evaluated *Linum* genomes are notably contracted and heavily biased toward TNL subclasses (Figure 3a). Within wild *Linum* species, active birth-and-death evolutionary processes drove dynamic copy number variations, ranging from a highly reduced repertoire in *L. grandiflorum* (144) to moderate expansions in *L. tenue* (345) and *L. perenne* (304).

Strikingly, despite this genus-wide evolutionary contraction, the specific domestication transition from the wild progenitor *L. bienne* to cultivated flax (*L. usitatissimum*) did not trigger a further quantitative genetic bottleneck (Figure 3a). Cultivated flax exhibits an exceptionally conserved macroscopic NLR architecture regardless of its agricultural ecotype. Both fiber (Bolchoi, 222; YY5, 219) and oil (Attila, 220; Marquise, 214; T397, 215) varieties maintain a nearly identical total number of NLRs compared to their wild ancestor *L. bienne* (217). Furthermore, their subclass distributions remain rigidly stable, typically comprising approximately 40 CNLs and 143–151 TNLs.

Although the global quantity of NLRs remained remarkably static during domestication, high-resolution spatial analysis revealed a prominent difference in the chromosomal assignment of a major NLR-rich region between *L. bienne* and cultivated flax assemblies (Figure 3b). A prominent NLR cluster was located on chromosome 2 in *L. bienne*, whereas the corresponding high-density NLR cluster in cultivated flax accessions was consistently observed on chromosome 14. Initially, this pattern appeared as a reciprocal presence-absence variation between the wild progenitor and cultivated accessions. However, to test whether this represented independent cluster loss and gain or a larger-scale chromosomal correspondence, we performed JCVI-based synteny analysis between *L. bienne* and T397.

The synteny analysis revealed clear collinearity between *L. bienne* chromosome 2 and T397 chromosome 14, including the genomic context surrounding the NLR-rich regions (Figure 3c). This result indicates that the apparent absence of the chromosome 2 cluster in cultivated flax and the presence of the chromosome 14 cluster in T397 should not be interpreted as unequivocal evidence for a *de novo* NLR burst in cultivated flax or complete cluster loss from *L. bienne*. Instead, the reciprocal pattern is more consistent with chromosome-level redistribution, large-scale rearrangement, or chromosome anchoring/assembly-configuration differences between the currently available assemblies.

### Transposable element dynamics drive genome size variation and recent bursts in the *Linum* genus

**FIGURE 4:**
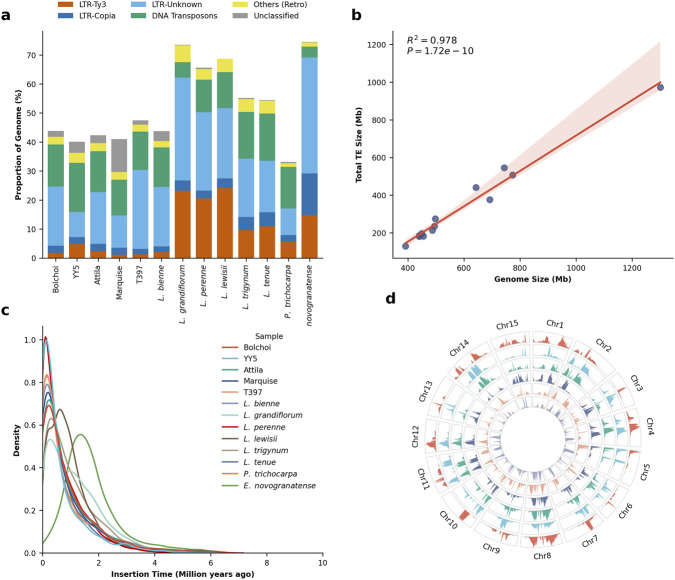
Repetitive sequence landscape and evolutionary dynamics of LTR retrotransposons in the *Linum* genus. **(a)** Stacked bar chart showing the proportions of major transposable element (TE) classifications across 11 *Linum* accessions and two outgroups relative to their genome sizes. LTR retrotransposons (Ty3, Copia, and Unknown) are the dominant components. **(b)** Linear regression analysis of total TE size against assembled genome size, demonstrating a robust positive correlation (*R* ^2^ = 0.978, *P* = 1.72 × 10^−10^). The shaded red area represents the 95% confidence interval. **(c)** Density plot illustrating the distribution of insertion times (in million years ago, Mya) for intact LTR retrotransposons. **(d)** Circos plot detailing the chromosomal distribution of LTR retrotransposons along the 15 pseudochromosomes. Tracks represent LTR density in sliding windows. Concentric tracks from outer to inner represent five cultivated *Linum usitatissimum* genomes (Bolchoi, YY5, Attila, Marquise, T397) and the wild progenitor *L. bienne*, highlighting the strong spatial conservation of LTR-rich pericentromeric regions among cultivated accessions and L. bienne, rather than indicating domestication-associated LTR amplification.

Transposable elements (TEs) are recognized as primary drivers of genome size evolution and structural variation in higher plants. To comprehensively characterize the repeat landscape within the *Linum* genus, we systematically annotated TEs across the 11 *Linum* genomes and two outgroups (*Populus trichocarpa* and *Erythroxylum novogranatense*) using the EDTA pipeline. The proportion of TEs varied substantially among the species, ranging from ∼33.1% in *P. trichocarpa* to over 70% in *L. grandiflorum* and *E. novogranatense* (Figure 4a). Within the *Linum* genus, Long Terminal Repeat (LTR) retrotransposons, notably the Ty3 and unclassified LTR superfamilies, constituted the major fraction of the repetitive sequences. Strikingly, wild species such as *L. grandiflorum* (23.13%), *L. lewisii* (24.21%), and *L. perenne* (20.51%) exhibited a massive expansion of Ty3 LTR retrotransposon elements compared to the cultivated flax (*L. usitatissimum*) accessions (∼1.06–4.85%). Linear regression analysis revealed a near-perfect positive correlation between total TE size and genome assembly size (*R* ^2^ = 0.978, *P* = 1.72 × 10^−10^) (Figure 4b), confirming that total TE content was strongly correlated with genome assembly size.

To uncover the evolutionary tempo of these TE amplifications, we estimated the insertion times of intact LTR retrotransposons. The density distributions of insertion times revealed distinct, species-specific temporal bursts of LTR activity (Figure 4c). Remarkably, the *Linum* genomes are characterized by highly recent LTR proliferation. Cultivated flax accessions (Bolchoi, YY5, Attila, Marquise, T397) and their immediate wild progenitor, *L. bienne*, displayed synchronous and extremely recent amplification peaks, with median insertion times occurring within the last half-million years (0.31–0.50 Mya), which substantially predate the onset of flax domestication approximately 8,000–10,000 years ago. In contrast, more distant wild relatives such as *L. grandiflorum* (0.97 Mya) and the outgroup *E. novogranatense* (1.54 Mya) exhibited relatively older amplification histories. This suggests a major LTR burst occurred shortly before or during the divergence of the *L. bienne*/*L. usitatissimum* lineage.

Finally, we mapped the chromosomal distribution of LTR retrotransposons across the 15 pseudochromosomes of five cultivated flax accessions and *L. bienne* (Figure 4d). The spatial distribution of LTRs is highly uneven, showing pronounced localized enrichments that typically correspond to pericentromeric and centromeric heterochromatin regions. Importantly, the genomic topography of these LTR-dense regions exhibits striking macrosyntenic conservation across all six analyzed genomes from the outside to the inside tracks (Bolchoi to *L. bienne*).

### Lineage-specific remodeling of fatty acid desaturase repertoires across *Linum* highlights copy-number divergence in the FAD2 and FAD3 families

To characterize fatty acid desaturase repertoires across Linum, we first identified membrane-bound desaturase candidates using the PF00487 domain and then classified FAD2 and FAD3 members by reference-guided phylogenetic analysis with *Arabidopsis* desaturases, including FAD2, FAD3, FAD6, FAD7, FAD8, FAH12, SLD1, SLD2, and ADS1. The initial HMM search recovered 20–49 PF00487-containing desaturase-like genes across the 13 genomes, with larger candidate sets in *L. usitatissimum* accessions and *L. bienne* than in the two outgroups. Phylogenetic classification separated the canonical FAD2 and FAD3 clades, which were used for subsequent copy-number and structural comparisons (Supplementary Table S6).

**FIGURE 5:**
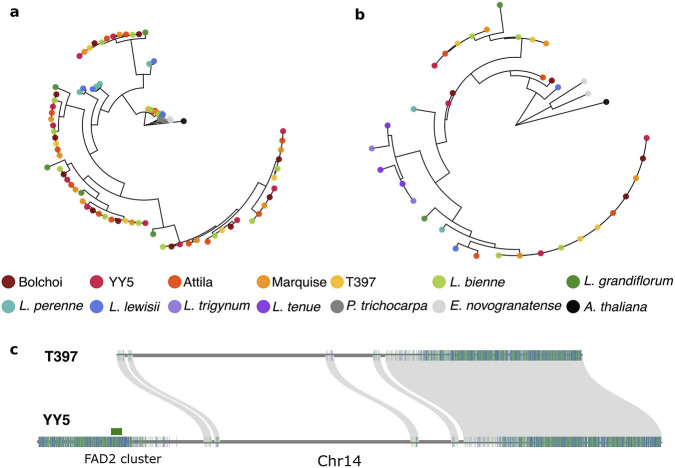
Evolutionary and structural variation of the FAD2 and FAD3 gene families across *Linum*. **(a)** Maximum-likelihood phylogeny of the FAD2 family identified from *Linum* species and outgroups following reference-guided classification with *Arabidopsis* desaturases. Expanded cultivated flax and *L. bienne* copies form multiple well-supported subclades corresponding to duplicated and tandemly amplified loci, whereas *L. grandiflorum*, *L. lewisii* and *L. perenne* retain reduced repertoires. Outgroup sequences from *Erythroxylum novogranatense* and *Populus trichocarpa* form smaller, separate lineages. **(b)** Maximum-likelihood phylogeny of the FAD3 family. In contrast to FAD2, FAD3 copy number is comparatively conserved across *Linum*, with four copies in *Linum usitatissimum* genomes and two to three copies in other species. *E. novogranatense* forms an external sister lineage, whereas no canonical *P. trichocarpa* sequence is recovered within the core FAD3 clade in this analysis. **(c)** Microsynteny comparison between YY5 and T397 across the FAD2-containing region. Vertical bars indicate annotated protein-coding genes and grey ribbons connect homologous loci between genomes. A putative large structural absence in T397 corresponds precisely to the interval harboring seven FAD2 genes in YY5, explaining the marked contraction of the FAD2 family in T397.

The FAD2 family showed substantial copy-number variation across the analyzed genomes (Figure 5a). Bolchoi, YY5, Attila, and *L. bienne* each contained 13 FAD2 genes, whereas Marquise contained 12 and T397 contained six. In these cultivated flax and *L. bienne* genomes, most FAD2 genes were organized in tandem arrays on two chromosome regions, with one region containing five to six tandem copies plus one isolated copy and the other showing a similar clustered arrangement. In T397, one FAD2-rich chromosomal region was absent, corresponding to the reduced copy number. Outside cultivated flax and *L. bienne*, FAD2 copy number was lower: *L. grandiflorum* contained six copies, *L. lewisii* and *L. perenne* contained four and five copies, respectively, and the two outgroups each contained two FAD2-like genes. No readily recognizable canonical FAD2 genes were recovered from *L. tenue* or *L. trigynum* in the reference-guided phylogenetic analysis.

Phylogenetic analysis showed that the expanded FAD2 copies in cultivated flax and *L. bienne* were grouped into two major chromosome-associated sets corresponding to chromosome 12-like and chromosome 14-like regions (Figure 5a). T397 lacked one of these phylogenetic subsets, matching the absence of the corresponding FAD2-rich region in the chromosomal distribution. Microsynteny comparison between YY5 and T397 further showed that the YY5 region containing seven FAD2 genes lacked a collinear counterpart in the corresponding T397 chromosome interval (Figure 5c). HiFi read-alignment analysis supported this pattern: T397 reads mapping to the YY5 FAD2-rich interval were accounted for by homologous regions retained on T397 chromosome 12 rather than by an unassembled T397 chromosome 14 segment. These results indicate that the reduced FAD2 copy number in T397 is unlikely to be caused solely by missed gene annotation or a simple assembly gap, although targeted experimental validation will be required to define the exact structural breakpoint.

To further evaluate the apparent absence of canonical FAD2 genes in *L. tenue* and *L. trigynum*, we performed genome-level TBLASTN searches using *Arabidopsis* FAD2 as the query. In YY5, used as a positive control, this search recovered all 13 FAD2 genes identified by phylogenetic classification. In *L. trigynum*, all retained TBLASTN hits overlapped PF00487-containing desaturase candidates, and no additional full-length FAD2-like locus was detected. In *L. tenue*, 14 of 15 retained hits overlapped PF00487-containing candidates, whereas the only non-overlapping hit was a short, low-coverage fragment and did not represent a full-length FAD2-like locus. Thus, no additional high-confidence canonical FAD2-like genomic regions were recovered outside the domain-supported candidate sets in these two species.

Compared with FAD2, the FAD3 family was more conserved in copy number and phylogenetic structure (Figure 5b). All five *L. usitatissimum* genomes retained four FAD3 copies distributed across three chromosomes in a 1+1+2 pattern. *L. grandiflorum*, *L. lewisii*, *L. perenne*, and *L. trigynum* each contained two FAD3 copies, whereas *L. tenue* contained three. The two *E. novogranatense* genes formed an external lineage relative to the *Linum* FAD3 clade, while no *P. trichocarpa* candidate was placed within the same canonical FAD3 clade as *Arabidopsis* FAD3 and the *Linum* FAD3 homologues in this analysis. Overall, FAD2 displayed greater copy-number and structural variation than FAD3 across the analyzed *Linum* genomes.

## Discussion

Accurate comparative genomics requires not only high-quality genome assemblies but also comparable gene annotation strategies (Stanke et al., 2006; Yandell and Ence, 2012; Salzberg, 2019). In this study, the original *Linum* annotations differed substantially in gene number and were generated using different pipelines and different levels of transcriptomic evidence. Some accessions were annotated with accession-specific RNA-seq data, whereas others relied on public transcriptomes from different genotypes, and original annotation files were unavailable for T397 and *L. bienne*. We therefore used ANNEVO as a unified annotation framework to reduce methodological heterogeneity across species. This strategy produced more compact gene sets while maintaining broadly comparable BUSCO completeness, and RNA-seq validation in YY5 showed stronger transcript support for ANNEVO gene models than for the original annotation. Nevertheless, we do not consider ANNEVO annotations to be a definitive replacement for fully evidence-based annotations. Rather, they provide standardized gene sets suitable for cross-species comparison under the current limitation that comparable multi-tissue RNA-seq resources are not available for all Linum species. Future genome projects integrating matched transcriptomic and proteomic evidence across species will further refine these annotations.

Based on this robust framework, our phylogenomic analysis inferred a profound macro-evolutionary gene family expansion occurring at the ancestral node shared by *L. bienne* and *L. usitatissimum*, likely preceding human domestication. This suggests that the genetic raw material potentially contributing to the diverse phenotypic radiation of flax was already present as standing variation in wild populations (Doebley et al., 2006; Olsen and Wendel, 2013). Following domestication, divergent artificial selection may have efficiently harnessed this redundancy (Lynch and Conery, 2000; Panchy et al., 2016). Fiber flax lineages showed enrichment of candidate lineage-associated gene families related to vesicle trafficking and cell wall biogenesis, processes relevant to polysaccharide deposition required for bast fiber elongation (Guerriero et al., 2017; Gorshkova et al., 2023). Conversely, oil flax accessions exhibited functional enrichment in photosynthetic components and fatty acid metabolism, consistent with possible breeding-associated changes in source-sink relationships to maximize carbon flow toward seed lipid accumulation (Baud and Lepiniec, 2010; Bates et al., 2012).

While domestication optimized developmental and metabolic traits, it also reshaped the genomic landscape of plant immunity (Michelmore and Meyers, 1998; Baggs et al., 2017). The *Linum* genus generally maintains a contracted, yet heavily TNL-biased, NLR repertoire compared to outgroups. However, the transition from *L. bienne* to cultivated flax did not trigger a further quantitative bottleneck; instead, the current assemblies reveal a major difference in chromosomal assignment of an NLR-rich region (Meyers et al., 2003; Van De Weyer et al., 2019). In the original chromosome-density comparison, the major NLR cluster appeared on *L. bienne* chromosome 2 but on chromosome 14 in cultivated flax accessions, suggesting a reciprocal cluster-level difference. However, the JCVI synteny analysis showed that *L. bienne* chromosome 2 is collinear with T397 chromosome 14 in the corresponding genomic context. Therefore, this pattern should not be interpreted as definitive evidence for independent NLR cluster loss in L. bienne or a *de novo* domestication-associated NLR burst in cultivated flax. Instead, the most conservative interpretation is that the NLR-rich region has experienced chromosome-level redistribution or differs in chromosome assignment among the current assemblies. Given the known history of genome duplication and extensive chromosomal rearrangement in flax and related *Linum* species (You et al., 2018), such assembly-level or karyotypic complexity is expected to complicate direct chromosome-by-chromosome comparisons. Thus, our results support the stability of the overall NLR repertoire during flax domestication, but the precise structural mechanism underlying the chromosome 2/chromosome 14 NLR cluster correspondence will require future validation using long-read, Hi-C, or pangenome-scale resources.

Parallel to gene family dynamics, transposable elements act as central architects of genome evolution (Bennetzen and Wang, 2014). Our analyses confirm that LTR retrotransposons dictate genome size variations across *Linum* (Vitte and Panaud, 2005; Lisch, 2013). Strikingly, the cultivated flax and its progenitor *L. bienne* share a synchronized and exceptionally recent peak of LTR insertion activity (<0.5 Mya), distinct from the older TE profiles of distantly related wild species. However, this peak occurred hundreds of thousands of years before the onset of human domestication and therefore should not be interpreted as a domestication-driven event. Instead, it likely represents ancestral or lineage-inherited transpositional activity in the *L. bienne*/*L. usitatissimum* lineage, which may have contributed to the genomic background upon which later domestication and breeding acted. Despite this pervasive transpositional activity, the spatial macro-synteny of LTR-dense pericentromeric regions remained largely conserved across *L. usitatissimum* genomes, highlighting a dual genomic strategy where localized transposable element bursts may have contributed to micro-evolutionary variation while maintaining macro-karyotypic stability.

In the context of specialized metabolism, the contrasting evolutionary profiles of FAD2 and FAD3 suggest that the flax oil pathway has been shaped by differential constraints on sequential desaturation steps (Arondel et al., 1992). FAD2, which controls the entry of oleic acid into the polyunsaturated fatty acid branch, is highly expanded and structurally labile across *Linum*, whereas FAD3, which catalyzes the downstream conversion of linoleic acid to α-linolenic acid, is comparatively stable. This asymmetry is biologically plausible. In oilseed species, FAD2 often represents a major control point for bulk seed fatty acid composition and is recurrently associated with copy-number change, neofunctionalization, tissue specialization, and domestication-related selection (Okuley et al., 1994; Dar et al., 2017). Our data extend this view by showing that, within *Linum*, FAD2 expansion is not merely species-wide but organized into conserved tandem blocks that have been differentially retained, lost, or truncated among lineages and even among cultivated flax accessions.

The near identity of many cultivated flax FAD2 paralogues to those in *L. bienne* implies that much of the expanded repertoire predates domestication and was inherited from the wild progenitor rather than generated *de novo* during crop improvement. From this perspective, domestication may have acted more strongly on retention, structural rearrangement, and expression partitioning of pre-existing duplicates than on their origin (Meyer and Purugganan, 2013). The T397 genome is especially informative because it demonstrates that substantial intraspecific remodeling can occur through a single large structural deletion removing an entire FAD2-rich block. Such chromosome-scale variation offers a parsimonious mechanism for rapid phenotypic shifts in oil composition and may help explain why some flax accessions differ sharply in fatty acid profiles despite overall genomic similarity. This also emphasizes the importance of pangenomic or multi-accession frameworks when linking candidate genes to agronomic traits in flax.

Finally, the apparent absence of canonical FAD2 in early-diverging species *L. tenue* and *L. trigynum* raises an intriguing evolutionary question. If confirmed, this pattern would imply either repeated loss, deep divergence, or structural divergence of canonical FAD2-like loci in these early-diverging lineages, suggesting that basal *Linum* lineages may have evolved alternative solutions for maintaining membrane and storage lipid unsaturation (Browse and Somerville, 1991). However, because membrane-bound desaturases can exhibit substantial sequence divergence outside catalytic motifs (Los and Murata, 1998), this observation warrants future functional transcriptomic validation. Regardless, the broader pattern is clear: the flax lineage combines strong structural conservation of FAD3 with exceptional FAD2 genomic plasticity, establishing a genetic architecture potentially capable of fine-tuning the balance between linoleic and α-linolenic acid production that defines the nutritional and industrial value of flaxseed oil.

An additional limitation of this study is that the analyzed genomes differ in assembly level and contiguity. Although BUSCO completeness was high for most assemblies, chromosome-scale resolution and local contiguity were not uniform across species (Supplementary Table S1). For example, cultivated flax genomes and several wild *Linum* genomes are chromosome-level assemblies, whereas *L. grandiflorum* and *L. perenne* are contig-level assemblies, and *L. lewisii* contains a relatively large number of scaffolds. These differences may influence local gene family recovery, chromosomal localization of clusters, TE annotation, and synteny interpretation. Therefore, chromosome-level comparisons, particularly those involving NLR-rich regions and FAD2-containing blocks, should be considered provisional until validated with comparable long-read, Hi-C, and pangenome resources.

## Conclusion

By establishing a unified, deep-learning-based annotation framework for 11 *Linum* genomes and two outgroups, this study dissects the complex genomic legacy of flax domestication and diversification. Our analyses demonstrate that the phenotypic divergence into fiber and oil flax may have exploited a pre-existing reservoir of duplicated genes inferred to have expanded in the ancestral wild lineage leading to *L. bienne* and *L. usitatissimum*, and were associated with candidate pathway-level enrichments related to cell wall formation in fiber flax and lipid/photosynthetic metabolism in oil flax. Cultivated flax maintained quantitative stability of the NLR immune receptor repertoire relative to *L. bienne*, while the major NLR cluster showed chromosome-level redistribution between *L. bienne* chromosome 2 and cultivated-flax chromosome 14. JCVI-based synteny indicates that this pattern is associated with collinear genomic regions and therefore should be interpreted cautiously as chromosomal reorganization or chromosome-assignment differences in the current assemblies rather than a confirmed *de novo* NLR burst. Crucially, the structural lability of the FAD2 gene family—contrasting with FAD3 conservation and exemplified by a large block deletion in oil accession T397—suggests that large-scale copy-number variation and presence-absence polymorphisms may contribute to variation in seed oil profiles, providing candidate targets for future pangenome-assisted breeding in flax.

## Data Availability

The original contributions presented in the study are publicly available. These data can be found in the NCBI Assembly repository under the accession numbers GCA_051167515.1, GCA_052935155.1, GCA_052935085.1, GCA_052935075.1, GCA_034768395.1, GCA_965225055.1, GCA_965366145.1, GCA_965231395.1, GCA_946122785.1, GCA_964030455.1, GCA_000002775.4, GCA_029891385.1., as well as in Zenodo at https://doi.org/10.5281/zenodo.4872894.
